# Derivation of feline vaccine-associated fibrosarcoma cell line and its growth on chick embryo chorioallantoic membrane – a new in vivo model for veterinary oncological studies

**DOI:** 10.1007/s11259-012-9535-9

**Published:** 2012-08-15

**Authors:** K. Zabielska, R. Lechowski, M. Król, K. M. Pawłowski, T. Motyl, I. Dolka, A. Żbikowski

**Affiliations:** 1 Department of Small Animal Diseases with Clinic, Faculty of Veterinary Medicine, Warsaw University of Life Sciences – WULS, Nowoursynowska 159c, 02-776 Warsaw, Poland; 2 Department of Physiological Sciences, Faculty of Veterinary Medicine, Warsaw University of Life Sciences - WULS, Nowoursynowska 159, 02-776 Warsaw, Poland; 3 Department of Animal Environment Biology, Faculty of Animal Sciences, Warsaw University of Life Sciences - WULS, Ciszewskiego 8, 02-786 Warsaw, Poland; 4 Department of Pathology and Veterinary Diagnostics, Faculty of Veterinary Medicine, Warsaw University of Life Sciences - WULS, Nowoursynowska 159, 02-776 Warsaw, Poland

**Keywords:** Feline vaccine - associated fibrosarcoma, Chorioallantoic membrane, Cell line derivation, Immunohistochemical examination

## Abstract

Feline vaccine associated fibrosarcomas are the second most common skin tumor in cats. Methods of treatment are: surgery, chemotherapy and radiotherapy. Nevertheless, the usage of cytostatics in feline vaccine associated sarcoma therapy is limited due to their adverse side effects, high toxicity and low biodistribution after i.v. injection. Therefore, much research on new therapeutic drugs is being conducted. In human medicine, the chick embryo chorioallantoic membrane (CAM) model is used as a cheap and easy to perform assay to assess new drug effectiveness in cancer treatment. Various human cell lines have different tumors growth on CAM. In veterinary medicine such model has not been described yet. In the present article derivation of feline vaccine associated fibrosarcoma cell line and its growth on CAM is described. The cell line and the tumor grown were confirmed by histopathological and immunohistochemical examination. As far as we believe, this is the first attempt to create such model, which may be used for further in vivo studies in veterinary oncology.

## Introduction

Feline vaccine-associated fibrosarcomas are the second most common kind of skin tumors found in cats. Predictive factors are vaccination against rabies and feline leukemia virus (FeLV) due to the aluminum adjuvant ions contained in these vaccines (Madwell et al. 2001; Couto et al. 2002). The exact mechanism of the formation of fibrosarcomas in cats is unknown, but it is suspected that the overexpressed inflammation of skin in the place of injection changes into neoplasm. According to the latest reports the genetic predisposition may also be important (Nambiar et al. 2001). It has been proven that cats injected many times in the same place (especially in the intrascapular region) are more likely to develop tumors. Thus, according to the newest guidelines published by the Vaccine Associated Feline Sarcoma Task Force from the U.S. cats should be injected near to pelvic limbs changing sides, because in severe cases leg amputations can be performed. Initially diagnosis is made based on the history, clinical examination of the patient and results of a fine-needle aspiration biopsy of the tumor. The definitive diagnosis is based on a histopathological examination of the tumor after its surgical resection. Methods of treatment are: surgery, chemotherapy and radiotherapy. Surgery is the first method of treatment; however, not all cancers can be removed surgically, depending on the type, the malignancy level, the localization and size of the tumor and above all the condition of the patient. The additional treatment is chemotherapy and radiotherapy. It has been proven that chemotherapeutic agents such as doxorubicin, cyclophosphamide and vincristine show high efficiency against soft tissue sarcomas, including feline vaccine associated fibrosarcomas. Unfortunately, the use of cytostatics in adjuvant therapy of feline vaccine-associated fibrosarcomas is limited due to their adverse side effects, high toxicity and low biodistribution after intra venous (*i.v*.) injection (MacDonald 2009). New drugs, as e.g. nanoparticles conjuncted with cytostatics are currently being tested (Brigger et al. 2002).

Animal models (most common rodent models) are used during preclinical in vivo studies. In human medicine, the chick embryo chorioallantoic membrane (CAM) model is also used as a cheap and easy to perform assay to assess a cancer’s ability to metastasis, pro- and anti- angiogenic potential and to assess new drug’s effectiveness (Dagg et al. 1956; Armstrong et al. 1982; Strum 1983; Ribatti et al. 2000; Vargas et al. 2007). As it has many advantages comparing with mouse model, we decided to create the first CAM model in veterinary medicine. The main advantage of this model is the ease of implementation and the faster tumor growth compared to other animal models (eg. on CAM it is 5–7 days, on rodent models about 3–6 weeks). Moreover, it is more ethical and a relatively cheap model and there is no need to keep animals. If experiments finish before hatching, according to the European law (Directive 2010/63/EU of the European Parliament and of the Council of 22 September 2010 on the protection of animals used for scientific purposes) the bioethical commission’s approval is not necessary. The only disadvantage is that the tumors exist for only 7–10 days (because the chick embryo development lasts 21 days and the tumor cells are usually implemented between the 6th and the 8th day of the chick embryo cycle). This model is well described for the human neuroblastoma cell lines (IMR 32) (Balke et al. 2010) and human osteosarcoma cell lines (HOS, MG63, MNNG-HOS, OST, SAOS, SJSA1, U2OS, ZK58) (Mangieri et al. 2009). It has been approved by the United States Food and Drug Administration (FDA) as an alternative method for preclinical testing. That model in veterinary medicine may simplify conducting in vivo studies, enable better knowledge about biology of various tumors and assess the effectiveness of different novel drugs.

Thus, the present study aimed to isolate a feline vaccine-associated sarcoma cell line and to cultivate it on CAM. We aimed to create a new model for further in vivo veterinary oncologic studies. As far as we believe, this is the first study concerning the use of CAM in veterinary medicine.

## Materials and methods

### Tumor sample

The tumor sample was obtained from an 8 years old feline patient with a history and clinical signs typical for vaccine-associated feline fibrosarcoma. The patient had been diagnosed for the recurrence of the vaccine-associated feline fibrosarcoma in the intrascapular region - the same place where the primary tumor was surgically removed 2 years before. The fine-needle aspiration biopsy confirmed the vaccine-associated fibrosarcoma. Blood tests and chest radiograph were performed. The blood results were in the reference value and no metastases to the lungs were found. The patient was classified for surgery and a surgical resection was performed according to the standard procedure. The histopathological examination of surgically removed tumor sample confirmed the previously given diagnosis (feline vaccine-associated fibrosarcoma).

### Isolation of the feline vaccine-associated fibrosarcoma cell line (FVAF1)

The procedure of cells isolation from tumor tissue has been described previously (Pawłowski et al. 2009; Król et al. 2010). Immediately after tumor resection the tumor was aseptically collected to the Dulbecco’s Modified Eagle Medium (DMEM) containing flask and was transported to the cell culture laboratory. The tumor sample was then sliced and cultured overnight in collagenase containing medium DMEM according to the Limon et al. protocol (Limon et al. 1986) (modified by Dr Eva Hellmen, Swedish University of Agricultural Sciences, Sweden). The following day, the medium was centrifuged and pellet was suspended in a fresh culture medium. The cells were cultured under optimal conditions: a DMEM enriched with 10 % (v/v) heat-inactivated fetal bovine serum (FBS), penicillin-streptomycin (50 IU mL–1), and fungizone (2.5 mg mL−1) (reagents obtained from Sigma Aldrich, USA), in an atmosphere of 5 % CO_2_ and 95 % humidified air at 37 °C, and routinely sub-cultured every other day.

### Histopathological and immunohistochemical examination

The tissue sample embedded in paraffin block was cut into 5 μm sections and baked in 37 °C overnight. After dewaxing in xylene and rehydration in ethanol, for antigen retrieval, the slides were placed in 0.02 M citrate buffer, pH 6.0 and boiled in the decloaking chamber. The tumor type was established based on the histopathological features of vaccine-associated feline sarcomas which have been previously described in the literature (Goldschmidt and Hendrick 2002; Madwell et al. 2001; Vascellari et al. 2003), whereas the tumor grading was based on the criteria proposed by Couto et al. (2002). The feline vaccine-associated fibrosarcoma cells (FVAF1) were cultured on Lab-Tek (Nunc Inc., USA) 4-chamber culture slides and were then fixed with ethanol after 24 h. The immunohistochemical examination of the expression of vimentin, smooth muscle actin, desmin and cytokeratin was performed on the tissue sample as well as on the FVAF1 cell line to confirm the same expression of antibodies in tumor cells as in the tissue sample. The usage of primary antibodies for different fibrosarcomas, including feline fibrosarcomas, both in cell lines and tissue samples, was described in many previous studies (Vascellari et al. 2003; Madwell et al. 2001; Goldschmidt and Hendrick 2002) and PhD thesis (“Untersuchungen zur Transkription von Wachstumsfaktoren und Zytokinen an felinen Vakzinationsstellen” Löhberg-Grüne, 2009; “Klonierung feliner Fibrosarkomzelllinien und deren zytogenetische Charakterisierung” Wasieri, 2009). The samples were incubated in the Peroxidase Blocking Reagent (Dako, Denmark) for 10 min at room temperature prior to the antibody incubation. After 30 min incubation in 5 % bovine serum albumin (Sigma Aldrich, Germany), the following primary antibodies were used (diluted in 1 % bovine serum): monoclonal mouse anti-human cytokeratin (Clone MNF116); monoclonal mouse anti-human vimentin (Clone Vim 3B4); monoclonal mouse anti-human actin (smooth muscle) (Clone 1A4); monoclonal mouse anti – human desmin (Clone D33) all antibodies at the concentration 1:50, obtained from Dako (Denmark). According to the manufacturer’s instructions the slides were incubated with antibodies at +4 °C overnight or 1 h at room temperature. For the staining the anti-mouse EnVision kits (Labelled Polymers consist of secondary anti-rabbit antibodies conjugated with the HRP enzyme complex obtained from Dako) was used. To develop the coloured product, the 3,3′-Diaminobenzidine (DAB) substrate was used (Dako). Finally, the haematoxylin was used for nuclei counterstaining. The staining without the use of primary antibodies was done as a negative control for each immunohistochemical analysis. The pictures were taken using Olympus microscopy BX60 (Olympus, Germany).

The tumor grade was established according to the grading scheme, previously adapted to the dog (Powers et al. 1995) and recently applied to feline vaccine associated fibrosarcomas (Couto et al. 2002; Vascellari et al. 2003), basing on cellular differentiation, presence and extension of necrosis within the neoplasm and mitotic rate.

### FVAF1 cell line growth on CAM

The new method of implementation of feline tumors cell cultures was developed by modifying the method of implementation of human tumors (eg. neuroma, osteosarcoma) in the chick embryos’ chorioallantoic membrane (Balke et al. 2010; Mangieri et al. 2009).

The 30 chick embryos (Ross 308 line, Pankowski Jan Poultry Hatchery, Poland) were held in the CO_2_ incubator (SMA Coudelou ZA 37210, France) under standard conditions (65 % humidity, 5 % CO_2_ and 37.5 °C) as soon as the embryogenesis started. On the 5th day of incubation a 5 mm × 5 mm ‘window’ was made in the eggshell on the blunt end on each of the egg. The parchment-like membrane was carefully taken out and a sterile silicon ring was put into CAM of each egg in accordance to aseptic procedures. Medium hard silicon rings, which are 7 mm in external diameter, 5 mm in internal diameter and 2 mm thick, were specially designed for this experiment and produced by Zegir PTHU (Poland). They were sterilized before use. The ‘window’ in the egg’s shell was closed using Polopor (Viscoplast, Poland) - a special adhesive tape with high air and water vapor. After 24 and 48 h eggs were candled to check the vitality and estimate the mortality associated with manual manipulation. The silicon rings were put into CAM 2 days before tumor cells inoculation to exclude the mortality of chick embryos caused by blood vessel damage and possible overexpressed inflammatory reaction due to putting silicon rings. Moreover, after those 2 days the silicon rings were more stable in the CAM as the blood vessels spread into it. On the 7th day of incubation FVAF1 cells (5 × 10^6^ cells in 25 μl of medium per egg) were administered into 20 chick embryos. 7 chick embryos were used as a negative control (inoculated only with 25 μl of medium (DMEM) per egg). At this day, chick embryo’s vitality was 85 % (1 out of 20 chick embryos were alive, 3 were dead probably due to mechanical manipulations connected with taking out the parchment-like membrane and putting silicon rings in CAM). Both tumor cells and medium were injected exactly into the silicon rings. Then, the ‘window’ in the eggshell were closed with the adhesive tape again. Eggs were candled 24 and 48 h later to check their survival. On the 12th day of incubation 17 out of 20 inoculated chick embryos were alive and were examined with the video otoscope (Welch Allyn MacroView™ Veterinary Otoscope 71032, USA) for tumor growth. However, no tumor growth was observed (phot.3). On the 16th day of the incubation 16 chick embryos were alive and tumor growth was observed. To confirm growth of the injected tumor cells, a histopathological examination of tumors was performed on the 18th day of the incubation, according to the procedures given above. We performed the histopathological examination on the day 18th day as the CAM started to degenerate at the day 18–19. Until the end of the experiment 5 out of 6 chick embryos used as a negative control with medium alone were alive and did not show any tumor grown.

## Results

### Histopathological and immunohistochemical analysis of the cell line FVAF1

**Fig. 1:**
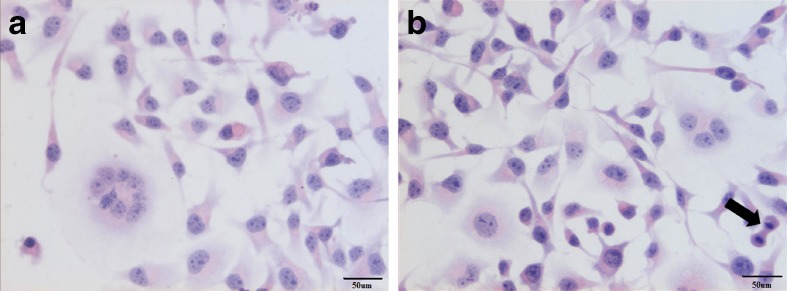
Feline vaccine-associated sarcoma cells stained with hematoxylin*-*eosin method. Multinucleated giant cells is visible on Fig. 1. original magnification 400×, and mitotic figure (*black arrow*)

**Fig. 2:**
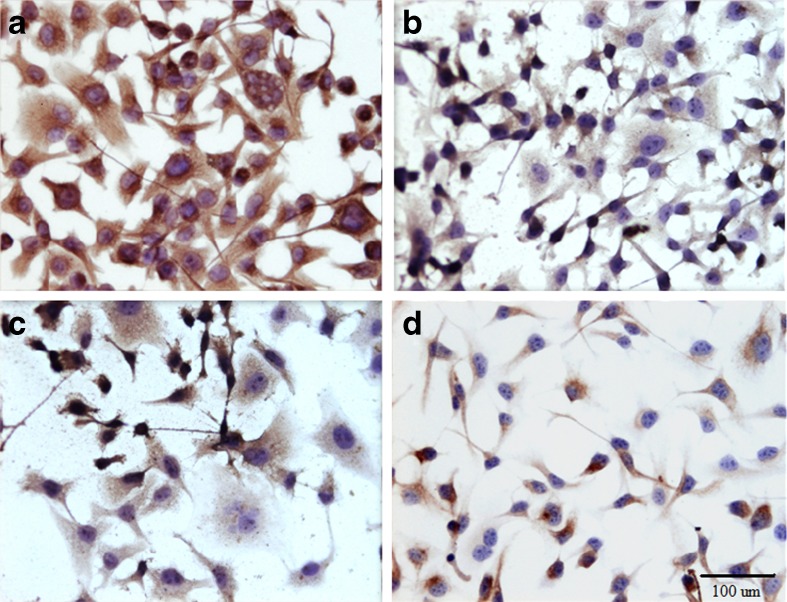
Feline vaccine-associated sarcoma cells stained for **a**) vimentin, note a strong cytoplasmic staining reaction (*brown color*), original magnification 400×, **b**) smooth muscle actin, original magnification 400×, **c**) desmin, original magnification 400×, **d**) cytokeratin, original magnification 200×. Peroxidase-based EnVision™kit (DakoCytomation, Denmark) and haematoxylin counterstain

The slides stained with haematoxylin*-*eosin showed the presence of multinucleated giant cells, pleomorphic cells with mild to marked atypia and mitotic figures. These findings appear to be most closely related to vaccine -associated feline sarcoma (VAFS) (Fig. 1a and b). Cell lines derived from vaccine-associated feline sarcoma were strongly positive for vimentin (+++) (Fig. 2a), some cells were positive for smooth muscle actin (+,+/−) (Fig. 2b), single cells positive for desmin (+,+/−) (Fig. 2c) and for cytokeratin (+/−) (Fig. 2d). Strong positive staining for vimentin confirms the mesenchymal origin of neoplastic cells.

### Tumor sample growth on CAM

#### Feline vaccine associated fibrosarcoma growth on chick embryo chorioallantoic membrane

**Fig. 3:**
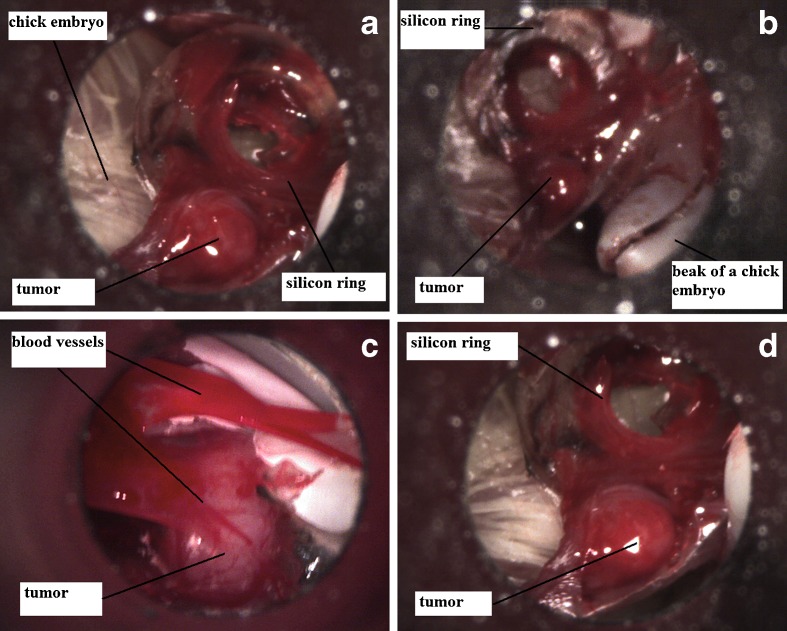
Feline fibrosarcoma on chick embryos on the 16th day of incubation (photographs taken by contractor, Welch Allyn MacroView™ Veterinary Otoscope 71032, USA)

The tumor growth was checked every 2 days since inoculation. Positive tumor development was reported when tumor angiogenesis was visible and when tumor size was of at least 2 mm in diameter. On the 16th day of incubation a tumor size of 5 mm (on average) in diameter was observed (Fig. 3a, b, c and d). The capillary vessels growing into the tumor were visible (Fig. 3c). The tumor had smooth surface, ovoid to spherical shape and was strongly attached to the CAM. It was located not exactly in the silicon ring, but a few milimeters out of it, either the tumor cells had spread through the vessels or the silicon ring position had slightly moved during embryogenesis and growth of the CAM and chick embryo.

#### Histopathological examination

**Fig. 4:**
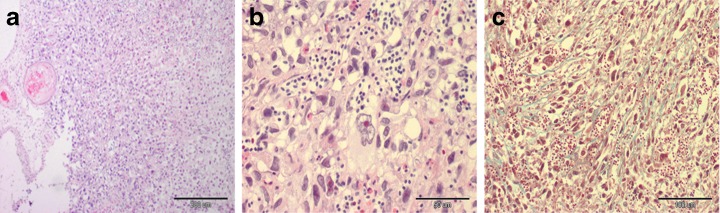
**a**) Microscopic examination of undifferentiated sarcoma. The neoplastic cells were arranged in an irregular pattern. Haematoxylin and eosin stain, original magnification 100×. **b**) Sarcoma characterized by neoplastic cells with atypia, eosinophilic cytoplasm and unclear cell border, some with prominent eosinophilic nucleoli. Haematoxylin and eosin stain, original magnification 400×. **c**) Interstitial collagen fibres (*green*) separated neoplastic cells were very scant*.* Masson’s trichome stain, original magnification 200×

The histopathological assessment of the tissue sample of small solid mass within CAM showed that the tumor was undifferentiated sarcoma (Fig. 4a and b). The mass was well demarcated, but non-encapsulated. Histologically, sarcoma consisted of pleomorphic, polyhedral, round or spindle-shaped cells with variations in size and shape of nuclei, nuclear hyperchromasia, prominent eosinophilic nucleoli, scant to abundant eosinophilic cytoplasm and indistinct cell borders. Some cells were bi- or trinucleated and some with eccentrically placed nuclei which resembled vacuolated cells. Neoplastic cells were mainly arranged without a specific pattern. Mitotic rate was low. Besides, the majority of neoformed vessels were present. No areas of necrosis within the tumor as well as no lymphocyte aggregates at the periphery of the mass were visible. Masson’s trichrome staining confirmed the mesenchymal components of this tumor and allowed to evaluate the amount and the distribution of collagen (Fig. 4c). According to the grading system on the basis of cellular differentiation, mitotic rate, presence and extension of necrosis, the sarcoma was classified as grade II.

#### Immunohistochemical examination

**Fig. 5:**
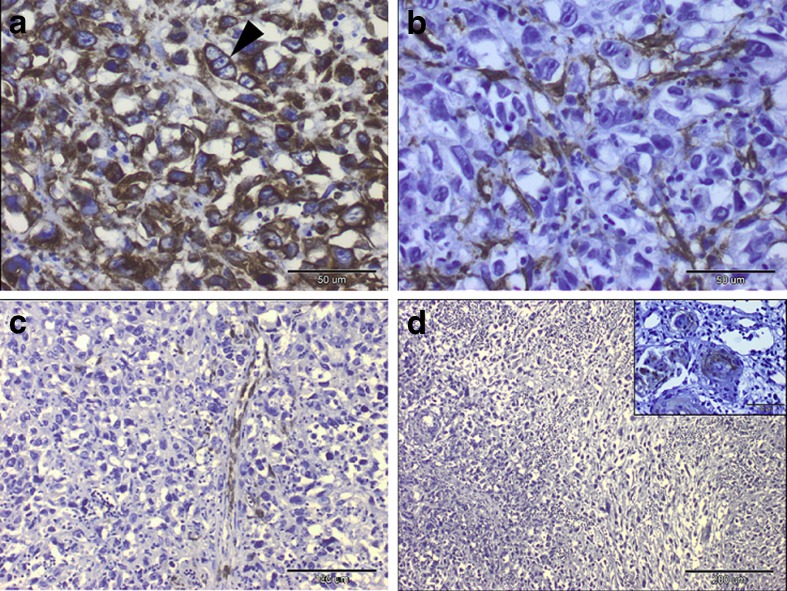
Immunohistochemical labelling of sarcoma: **a**. Intense vimentin-cytoplasmic immunostaining in neoplastic cells. Arrowhead showed a trinucleated neoplastic cell. Peroxidase-based EnVision™kit (DakoCytomation, Denmark) and hematoxylin counterstain, original magnification 400×. **b**. Cytoplasmic positivity of alpha-smooth muscle actin noted in some neoplastic cells (graph on the left) Peroxidase-based EnVision™kit (DakoCytomation, Denmark) and hematoxylin counterstain, original magnification 400×. **c**. Immunopositivity for desmin distributed among blood vessels. Peroxidase-based EnVision™kit (DakoCytomation, Denmark) and hematoxylin counterstain, original magnification 200×. **d**. No immunoreactivity with cytokeratin. Inset: Cytokeratin immune-staining were detected only in small foci of presumptive squamous epithelial cells within chorioallantois membrane of chicken embryo. Peroxidase-based EnVision™kit (DakoCytomation, Denmark) and hematoxylin counterstain, original magnification 100×, inset: 400×

Immunohistochemical examination delineated the mesenchymal components of tumor. The samples were strongly positive for vimentin (Fig. 5a). Some neoplastic cells were stained with smooth muscle actin antibody. Additionally, anti-actin and anti-desmin antibody labelled the walls of blood vessels (Fig. 5b and c). Neoplastic cells did not react with cytokeratin, which confirmed the non-epithelial origin of these cells (Fig. 5d). Only some foci of presumptive squamous epithelial cells showed cytokeratin expression.

According to the Couto et al. grading system (Couto et al. 2002) the tumor was classified as grade II of malignancy.

## Discussion

The only available commercial cell line of feline fibrosarcoma is from NBL Cell Line Collection – a part of American Type Culture Collection (ATCC, designation FC 77.T) and cannot be delivered to European countries (distributed only within the United States). That is the reason why the authors decided to isolate their own feline vaccine associated fibrosarcoma cell line (FVAF1), which gives the possibility to further investigate on molecular level and to find new methods of treatment.

Comparing the proposed model to rodent model the main advantage of the CAM model is the fact that the experiment can be repeated many times and a large amount of chick embryos may be used in one time, which gives more reliable results. It results from the fact that it is an in vivo animal model that does not require the approval of a bioethic committee in European countries. Performing the same number of experiments and the same number of species on rats would be rather impossible not only due to animal law regulation but also for ethical reasons. Moreover, the tumor growth in the presented model lasts 10 days, while on rodent model from 3 to 6 weeks. Relatively fast tumor growth makes this model easier to utilise.

Furthermore, it has the cognitive value showing possibility of feline vaccine associated fibrosarcoma growth on CAM. According to the available data in the field, human cancer cells growth on CAM are usually visible after 5–7 days after inoculation (on the 12th–14th day of incubation). In the animal model tumors were firstly visible after 9 days (on the 16th day of incubation) (Balke et al. 2010; Mangieri et al. 2009), what may suggest a slower growth of this particular cell line. The new element of the procedure (our modification) was the use of a video otoscope, which allowed visualization of the tumor growth relatively noninvasively without enlarging the ‘windows’ made in the egg’s shells and without too extensive embryo manipulations. This technique has been developed by our team and it has been considered as highly effective and minimally invasive method in comparison to other methods described in subject literature of checking the growth of human cell lines in chick embryo chorioallantoic membrane using a microscope with an external source of light (Balke et al. 2010). The literature about CAM model show many limitations with observation and photograph documentation of the tumors growth using standard microscope with external source of light. In many cases it is impossible to make photographs without enlarging the “window” in the egg shell as during embryogenesis silicon rings and tumors are not always visible in the middle of the window. On the other hand, in screening experiments contractors have checked that enlarging the “window” in the egg shell increases the mortality of chick embryos, probably due to the higher water permeability making chick embryos dry out. The video otoscope enables the observation of all tumors despite their localization without enlarging the “window” in the egg shell. There are some problems with making high quality photographs of 3D living organisms, but that does not depend on the kind of equipment used.

The biggest difficulty that the contractors had to deal with while performing screening studies was the high mortality of chick embryos connected with taking out the parchment-like membrane. Silicon rings were put on CAM on different days of embryogenesis (5th, 6th, 7th, 8th), noticing the lowest mortality at 5th day (around 10 %). The highest mortality was at 8th day reaching nearly 60 %, what was probably due to mechanical manipulations connected with easier damaging more developed blood vessels of CAM. This model, when performed before 11th day of chick embryo incubation, is available for xentographs as until this day the immune system of the chick embryo is immature

As far as we believe, our experiment of the implantation of the animal cell line on CAM was a first such study in the field of veterinary oncology. However, further studies using different cell lines are required to use this easy model for investigation of cancer biology and to assess the effectiveness of new anticancer drugs. Animal models are essential for entering new therapeutic agents into the market and can not be avoided. We believe that showing the possibility of first feline fibrosarcoma cell growth on CAM will encourage other veterinary scientists to further investigate using other animal cell lines and may be a basis to creating an inexpensive and relatively easy to use new experimental model.
